# Enteroptosis

**Published:** 1901-08

**Authors:** 


### ENTEROPTOSIS.

The investigations of Glenard, reported in 1885, and more recently those of J. Clarence Webster and Godart-Danheiux, have well established the important fact that the complex of symptoms to which it has become the fashion to refer as "neurasthenia," and for which often no satisfactory cause can be discovered, are in many instances dependent upon a displacement of certain of the abdominal viscera, notably the right kidney and transverse colon. To this condition the term *enteroptosis,* or *Glenard's disease,* has been given. The visceral displacement is dependent upon a relaxation of the abdominal wall, which by lowering intra-abdominal tension permits the contained viscera to drag upon their suspensary attachments. The chief causative factor as might be expected is frequent child-bearing, especially in those women who wear tight corsets or who do heavy work during the latter months of pregnancy, or, very often, soon after confinement. The symptoms commonly present are those of nervous dyspepsia, with more or less obstinate constipation and dull aching pains in the lumbar and iliac regions. The patient becomes pale, anemic and low spirited, and may present practically every symptom of so-called neurasthenia. Dr. Webster suggests that separation of the recti with stretching of the linea alba rather than a general relaxation of the abdominal fascia and muscles is responsible for the lowered tension, and he demonstrates this by placing the woman in a reclining position and pressing the finger tips of one hand into the space between the recti and then having her attempt to raise her head and shoulders. The muscles are thus put on the stretch and the degree to which they are separated estimated. Displacement of the various organs can usually be demonstrated by the ordinary means.

Enteroptosis is a fairly common condition, and when recognized early, much can be done for its relief. Its symptoms are most distressing, and it is a matter for regret that so many of the profession fail to recognize the definite physical basis for their occurrence, and therefore fail to institute any rational line of treatment. Although a cure can rarely be obtained except by operative measures, yet much relief always follows the judicious use of means to overcome constipation, abdominal massage, and the wearing of a well fitting abdominal supporting bandage. The subject is one of great importance, and one that directly concerns the general practitioner. R.

